# Supplementary material for: Transcriptional Dysregulation and Impaired Neuronal Activity in FMR1 Knock-Out and Fragile X Patients’ iPSC-Derived Models
Source: Int J Mol Sci. 2023 Oct 5;24(19):14926. doi: 10.3390/ijms241914926 (PMC10573568; doi:10.3390/ijms241914926)
Supplement: Supplementary file 1 [file ijms-24-14926-s001.zip › ijms-2614942-supplementary.pdf]

International Journal of *Molecular Sciences*

MDPI

Table S1. Nucleotide sequences used for Cripr editing, digital PCR, and Sanger sequencing.

**ssODN template containing the stop tag (in red)**	**TGCCACAAAAGATACTTTCCATAAGATCAAGCTGGATGTGCGTAAC TAGCTGA AGTTGATACACAAGAAATGCTGAGAACTTGGAAGTGATAT**
ddPCR primers	FMR1taqF: GAACGTCTAAGATCTGTTAATCCCA<br>FMR1taqR2: CCATTTTTGCCAAAGTCCACCA
LNA probes by IDT	FMR1wt-HEX: /5HEX/CCA +GAA +GA+C T+TA +CG/3IABkFQ<br>Stoptag-FAM: /56-FAM/CGTAA+C+TA+G+C+T+GA/3IABkFQ
Primers for PCR/Sanger sequencing	PCR<br>Forward: GAACGTCTAAGATCTGTTAATCCCA<br>Reverse: CCATTTTTGCCAAAGTCCACCA<br>Sequencing<br>GAACGTCTAAGATCTGTTAATCCCA<br>CCATTTTTGCCAAAGTCCACCA

Table S2. List of antibodies.

Cell type .	Antibody	Catalog	Dilution
**iPSC**	Nanog	Abcam Ab21624	1:200
	TRA1-60	Stem Cell Technologies #60064	1:200
	OCT3/4	Santa Cruz SC-8628	1:500
	SSEA-4	Santa Cruz SC-21704	1:500
**cNPC**	Nestin	Abcam ab92391	1:500
	SOX1	R&D AF3369	1:100
	Foxg1	Abcam ab196868	1:500
	Ki67	BD 556003	1:200
**Astrocytes**	GFAP	Millipore AB5804	1:250
**Cortical neurons**	Tuj1	Millipore MAB5564	1:200
	Tuj1	Covance	1:1000
	Map2	Encor Biotechnology AB_2138173	1:1000
	VGLUT1	Synaptic System 135304	1:200
	GABA	Sigma a2052	1:200

Table S3. List of TaqMan probes and primer sets (Applied Biosystems).

Gene	reference
OCT3/4	Hs04260367_gH
Nanog	Hs02387400_g1
FMR1	Hs00924547_m1
PAX6	Hs01088114_m1
SOX1	Hs01057642_s1
Nestin	Hs04187831_g1
MAP2	Hs00258900_m1
S100B	Hs00902901_m1
FOXG1	Hs01850784_s1
SIX3	Hs00193667_m1
MAP1B	Hs01067016_m1
GRIA2	Hs00181331_m1
GABRA2	Hs00168069_m1
SNAP25	Hs00938957_m1
SYN1	Hs00199577_m1
PSD95	Hs01555373_m1
TUBB3	Hs00964962_g1
GFAP	Hs00909233_m1

VIM	Hs00958111_m1
SYP	Hs00300531_m1
SLC17A7	Hs00220404_m1
NTRK2	Hs00178811_m1
GRIN2B	Hs01002012_m1
ACTB	Hs01060665_g1
GAPDH	Hs02786624_g1

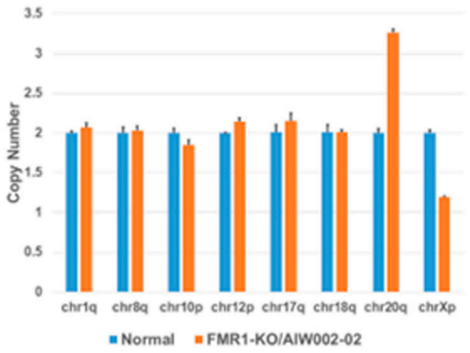

Figure S1. Genomic stability testing of the *FMR1* KO iPSC line. qPCR-based chromosomal abnormality analysis in the eight common critical areas within chr1q, chr8q, chr10p, chr12p, chr17q, chr18q, chr20q, and chrXp showed amplification in chr20q only.

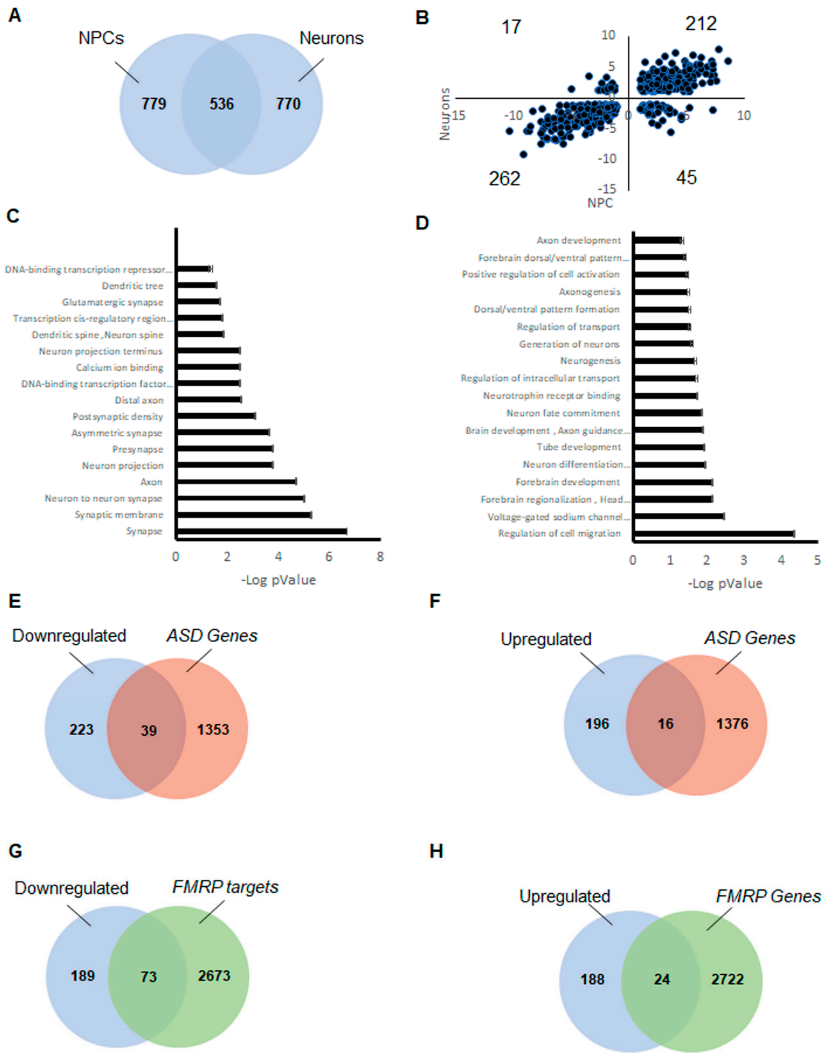

Figure S2. Common transcriptional deregulations between *FMR1*-KO NPCs and Neurons. A) Venn diagram of the differentially expressed genes in *FMR1* KO NPCs and *FMR1* KO neurons. B) Fold change distribution for the common set of genes differentially expressed in *FMR1* KO NPCs and Neurons. (C) GO terms defining the significant functional enrichment for the genes that are downregulated in *FMR1* KO NPCs and *FMR1* KO neurons. (D) GO terms defining the significant functional enrichment for the genes that are upregulated in *FMR1* KO NPCs and *FMR1* KO neurons. (E) Overlap between the genes significantly downregulated in *FMR1* KO NPCs and neurons, and the list of autism risk genes analyzed. (F) Overlap between the genes significantly upregulated in *FMR1* KO NPCs and neurons, and the list of autism risk genes. (G) Overlap between the genes significantly downregulated in *FMR1* KO NPCs and neurons, and the list of FMRP targets analyzed. (H) Overlap between the genes significantly upregulated in *FMR1* KO NPCs and neurons, and the list of FMRP targets analyzed.

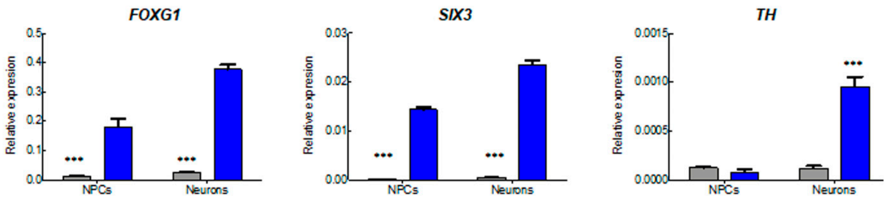

Figure S3. Validation of differential gene expression by Real-Time PCR. Normalized expression levels of *FOXG1*, *SIX3*, and *TH* mRNA in *FMR1* KO NPCs and neurons compared to control cells.

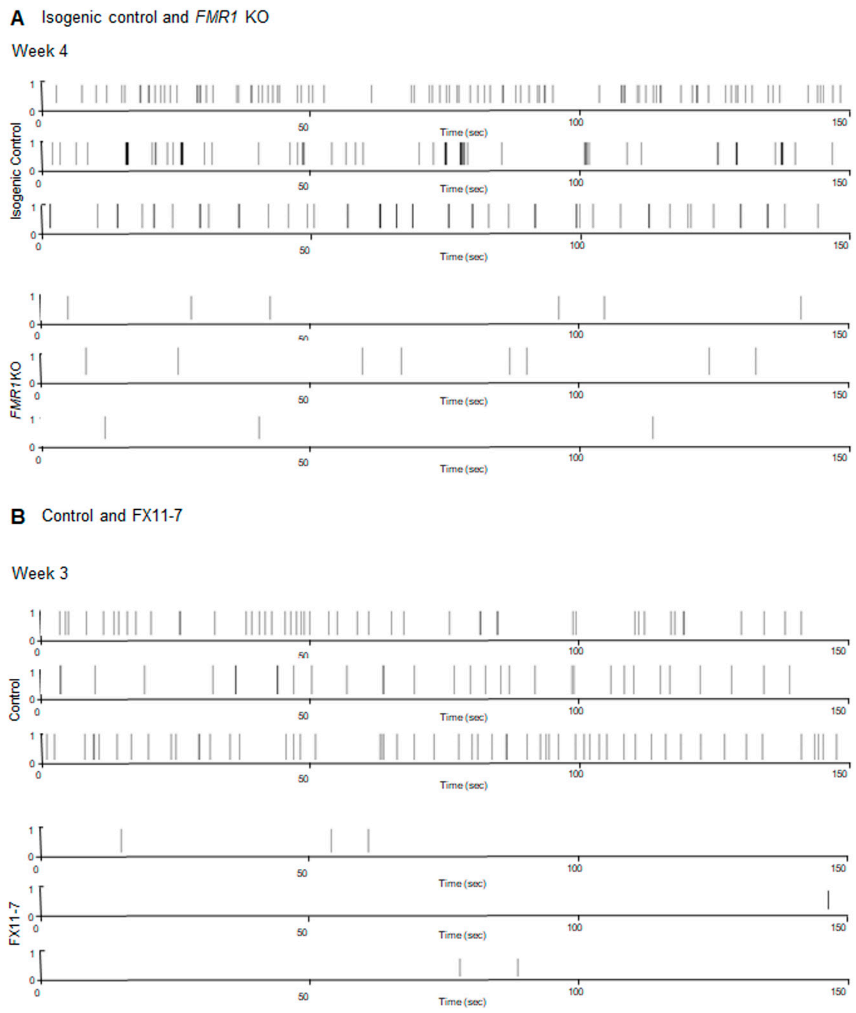

Figure S4. Validation of differential gene expression by Real-Time PCR. MEA raster plots from MEA recordings showing. Changes in electrical activity in week 3 to week 4 in cortical neurons from (A) Isogenic control and *FMR1* KO, and (B) Control and FX11-7.

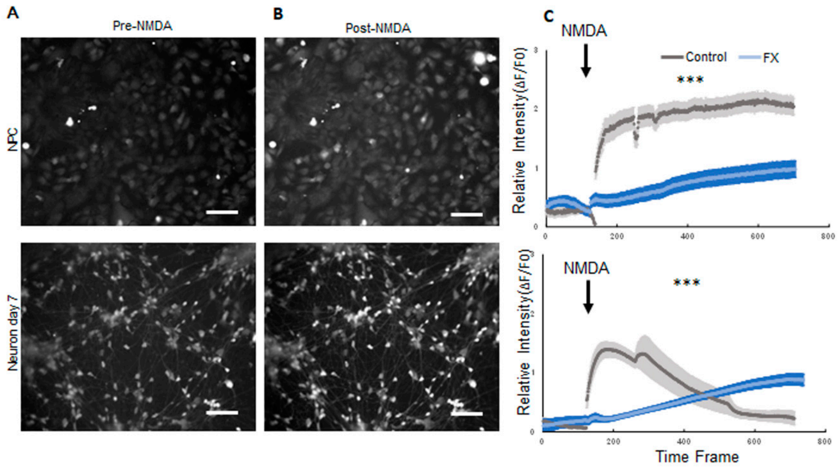

Figure S5. Differential NMDA response between control and FXS IPSC-derived NPCs and Neurons

Supplementary results

Common deregulations between FMR1 KO NPCs and FMR1 KO neurons.

The analysis of our RNA sequencing data shows that a similar number of genes were differentially expressed at the NPC and differentiating neuron stages (**Supplementary Figure 2**). Those genes include genes associated with synapse and neuronal formation, autism-associated genes, and FMRP targets. We then wondered how common the deregulated genes in *FMR1* KO cell lines at the NPC and neuronal stages are. Comparing the two gene lists **(Figure 6A and Figure 6B**), we found a set of 536 genes (40% of the DEGs) that were deregulated at both the NPC and neuronal stages (**Supplementary Figure 2**). We further examined the fold changes in the list of commonly deregulated genes. We found that 88% of them were either up or downregulated at both stages (**Supplementary Figure 2**). Genes that were found upregulated in *FMR1* KO NPCs and neurons are involved in brain development, neurogenesis, and forebrain specification (**Supplementary Figure 2**). These genes include *SIX3* and *FOXG1* which code for transcription factors whose mutations are respectively responsible for holoprosencephaly/schizencephaly [1], Rett-like, and FOXG1 [2] syndromes. We have actually validated by Q-PCR the increased expression of *SIX3* and *FOXG1* in *FMR1* KO NPCs and Neurons ($F_{SIX3\ Genotype}$=1145; df=1; p<0.0001; t_{NPCs}=18.27; p<0.001; $t_{Neurons}$=29.60; p<0.001; $F_{FOXG1\ Genotype}$=264.4; df=1; p<0.0001; t_{NPCs}=7.491; p<0.001;$t_{Neurons}$=15.50;p<0.001). Genes downregulated in the *FMR1* KO NPCs and neurons were significantly enriched in synaptic function, axon, neuronal projections, and DNA binding transcription factor (**Supplementary Figure 2**). These functional enrichments observed from common deregulations in *FMR1* KO NPCs and neurons suggest that the regional specification of *FMR1* KO cells and their neuronal differentiation are impaired, leaving the cells in a progenitor-like state. We have also assessed these gene lists for enrichment in ASD-related genes and FMRP targets. Similarly, to what was observed in **Figure 6**, we found significant enrichment in ASD-related genes (39/262; p < 9.309e-05) (**Supplementary Figure 2E**) and in FMRP targets (73/262; p < 2.526e-07) (**Supplementary Figure 2G**) among the list of genes that are commonly downregulated in *FMR1* KO NPCs and neurons.

References

1. Hehr, U.; Pineda-Alvarez, D. E.; Uyanik, G.; Hu, P.; Zhou, N.; Hehr, A.; Schell-Apacik, C.; Altus, C.; Daumer-Haas, C.; Meiner, A.; Steuernagel, P.; Roessler, E.; Winkler, J.; Muenke, M., Heterozygous mutations in SIX3 and SHH are associated with schizencephaly and further expand the clinical spectrum of holoprosencephaly. *Hum Genet* **2010**, 127, (5), 555-61.
2. Jacob, F. D.; Ramaswamy, V.; Andersen, J.; Bolduc, F. V., Atypical Rett syndrome with selective FOXG1 deletion detected by comparative genomic hybridization: case report and review of literature. *Eur J Hum Genet* **2009,** 17, (12), 1577-81.
